# Expression of Proteins, Glycoproteins, and Transcripts in the Guts of Fasting, Fed, and *Trypanosoma cruzi*-Infected Triatomines: A Systematic Review

**DOI:** 10.3390/pathogens12091124

**Published:** 2023-09-02

**Authors:** Olivia A. Reynoso-Ducoing, Berenice González-Rete, Elsa Díaz, Frida N. Candelas-Otero, J. Antonio López-Aviña, Margarita Cabrera-Bravo, Martha I. Bucio-Torres, Elia Torres-Gutiérrez, Paz María Salazar-Schettino

**Affiliations:** Departamento de Microbiología y Parasitología, Facultad de Medicina, Universidad Nacional Autónoma de México, Coyoacán, México City 04510, Mexico; oard@unam.mx (O.A.R.-D.); bere.gonzalez.ciencias@gmail.com (B.G.-R.); elsa1@ciencias.unam.mx (E.D.); fridanoelly@ciencias.unam.mx (F.N.C.-O.); anton_avina@ciencias.unam.mx (J.A.L.-A.); imay@unam.mx (M.C.-B.); marbu@unam.mx (M.I.B.-T.)

**Keywords:** triatomine, proteins, transcripts, glycoproteins, *Trypanosoma cruzi*, gut, intestine, Chagas disease, vectors

## Abstract

Chagas disease is caused by the hemoflagellate protozoan *Trypanosoma cruzi.* The main transmission mechanism for the parasite in endemic areas is contact with the feces of an infected triatomine bug. Part of the life cycle of *T. cruzi* occurs in the digestive tract of triatomines, where vector and parasite engage in a close interaction at a proteomic–molecular level. This interaction triggers replication and differentiation processes in the parasite that can affect its infectivity for the vertebrate host. With the aim of compiling and analyzing information from indexed publications on transcripts, proteins, and glycoproteins in the guts of fasting, fed, and *T. cruzi*-infected triatomines in the period 2000–2022, a systematic review was conducted following the PRISMA guidelines. Fifty-five original research articles retrieved from PubMed and ScienceDirect were selected; forty-four papers reported 1–26,946 transcripts, and twenty-one studies described 1–2603 peptides/proteins.

## 1. Introduction

Chagas disease, caused by the protozoan *Trypanosoma cruzi (Kinetoplastida,* Trypanosomatidae) (Chagas, 1909), considered by the World Health Organization (WHO) to be the most frequent parasitic disease in Latin America, has reached non-endemic countries in the Americas and the world in recent decades [1]. An estimated 6 million individuals in the world are infected with *T. cruzi*, with an incidence of 30 thousand new cases and a mortality rate of 12 thousand cases. Currently, more than 70 million people are at risk of acquiring the infection, mostly including poor and marginalized populations [2].

The disease is endemic in 21 countries in Latin America and the southern United States, where the most important transmission route for humans and other mammals involves contact with triatomine feces, the so-called vector transmission route. Triatomines are hematophagous insects of the subfamily Triatominae (Hemiptera: Reduviidae), known as “kissing bugs” or “vinchucas”; they generally feed at night on vertebrate blood. Defecation takes place during or within minutes after feeding. Infection occurs via the active penetration of the infective form (metacyclic trypomastigotes) through skin and mucous membranes [3].

The digestive system of a triatomine is formed by three sections. The section where food first arrives is the anterior midgut (AMG); it is also known as promesenteron, stomach, gut, or crop, and this is where the storage, concentration, and pre-digestion of ingested blood take place. In the posterior midgut (PMG), also known as the small intestine, postmesenteron, or midgut, nutrient digestion and absorption occur. The last section is the rectum (RE), proctodeum, rectal ampulla, or hindgut; some nutrient absorption occurs here, as well as waste excretion. If the insect is infected with *T. cruzi*, this is where metacyclic trypomastigotes leave the vector to continue their life cycle in a vertebrate host [4]. Various studies have focused on the perimicrovillar membrane (PMM), originally described by Lane and Harrison (1979). It is a structure of the triatomine PMG that grows upon food stimulus [5]. The PMM protects gut cells against enzymes and microorganisms found in ingested blood [6].

When a triatomine ingests blood from an infected vertebrate, the food migrates through the digestive system, where a very close interaction takes place, with recognition between surface components of erythrocytes, the parasite, and microbiota with those of the insect’s digestive epithelium. *Trypanosoma cruzi* is exposed to stressors such as changes in temperature, osmolarity, and pH, as well as to oxidative and proteolytic stress; thus, it interacts with its host, activating adaptive mechanisms and modulating the intestinal environment to move into the PMG. Epimastigote anchorage occurs in the PMM, where the parasite replicates by binary fission, thus maintaining the infection [4,7]. Finally, some parasites that pass into the RE undergo differentiation to the vertebrate-infecting form (metacyclogenesis). These processes are critical in the life cycle of the protozoan and have a significant impact on the transmission and infectivity of the parasite to the mammal [4,8]. In this regard, proteins and glycoproteins synthesized by the insect, mainly digestive enzymes (hemagglutinins and hemolysins) and immune response proteins (antimicrobial peptides), have been studied.

Several authors have reported specific interaction sites between *T. cruzi* and the different sections of the insect gut, which allow recognition, anchoring, reproduction, and differentiation to occur, allowing the parasite to maintain its life cycle [9,10,11,12].

Upon its publication, the genome of *Rhodnius prolixus* (Stål, 1859) has become a crucial tool to study the genetic information of these organisms during their life cycle, including their development, adaptation, and reproduction [13]. However, detecting expressed genes and gene products that perform cellular functions in an organism under a certain environmental condition has required the development of specialized techniques for the detection of RNA transcripts, peptides, and proteins.

For triatomines, the molecules involved in the interactions between the insect digestive tract and the parasite are of interest, as are the extrinsic and intrinsic factors that impact its vectorial capacity as a transmitter of *T. cruzi* to a mammalian host. Thus, knowledge on proteins and transcripts expressed in the digestive tracts of triatomines under different feeding and infection conditions is of great interest. This review is aimed at compiling and analyzing information from selected publications on transcripts, proteins, and glycoproteins from the guts of triatomines, as well as on any changes in their expression in different gut sections and under various feeding and infection conditions.

## 2. Materials and Methods

### 2.1. Systematic Search

In this study, a systematic review of the scientific literature on the expression of transcripts and proteins in the digestive tracts of Chagas disease vectors was conducted. A search was carried out in May 2022 in the PubMed (www.ncbi.nlm.nih.gov/pubmed; Accessed on 26 June 2023) and Science Direct (www.sciencedirect.com/; Accessed on 26 June 2023) databases, limiting original research publications to the period 2000–2022. The terms used were “Triatominae” OR “triatomine”; “proteome” OR “protein” OR “peptide” OR “protein expression”; and “gut” OR “digestive tract” OR “midgut”. A total of 110 results were obtained from PubMed and 35 from Science Direct. The guidelines of the PRISMA statement [14] for the correct conduction of systematic reviews were followed.

### 2.2. Inclusion Criteria

Original research articles published in indexed journals were included if they met the following criteria: (1) to refer to the triatomine species studied; (2) to indicate the region of the digestive tract analyzed (AMG, PMG, and/or RE); (3) to use one or more transcripts or protein detection techniques.

### 2.3. Information Capture

For each publication analyzed, the following data were recorded: year of publication, author, article title, and DOI (extracted by the Zotero bibliography manager (https://www.zotero.org/; Accessed on 26 June 2023); triatomine species, developmental stage, and sex; food source and infection status with *T. cruzi*; strain specification or isolate (DTU); gut sections studied; proteins expressed and detected in the gut, and transcript detection.

### 2.4. Parameters for Transcript, Protein, and Glycoprotein Analysis

The information was captured in tables that included the feeding and infection status of the insect, as well as the gut section where detection was performed. Changes in expression levels with feeding and infection conditions, and between different sections of the gut, were analyzed and reported if appropriate. 

In articles reporting transcript databases, transcript identifications reporting significant differences between intestinal sections, feeding conditions, and infection (either in the main text, supplementary figures, or tables) were collected. In the case of Ribeiro (2014), only transcripts reporting a difference greater than 10 arbitrary units between the gut sections studied were extracted.

As for the proteins, the total number of proteins reported in each included article was summarized. However, only those proteins discussed by the authors of each original article in their own text were analyzed. Some authors reported the identification of detected proteins in supplementary tables. The URLs of these supplementary tables are listed in the Supplementary Table S2 of Protein Compendium of this review. Alves et al. (2007) only provided the molecular weights of glycoproteins involved in the interaction between PMM and *T. cruzi* [12]. In the work by Gutiérrez-Cabrera et al. (2019), information on molecular weight was obtained from figures of electrophoretic runs with glycoprotein recognition by lectins [15]. Carbohydrate-free proteins were identified after Coomassie blue staining.

## 3. Results

### 3.1. General Analysis of Selected Articles

**Figure 1:**
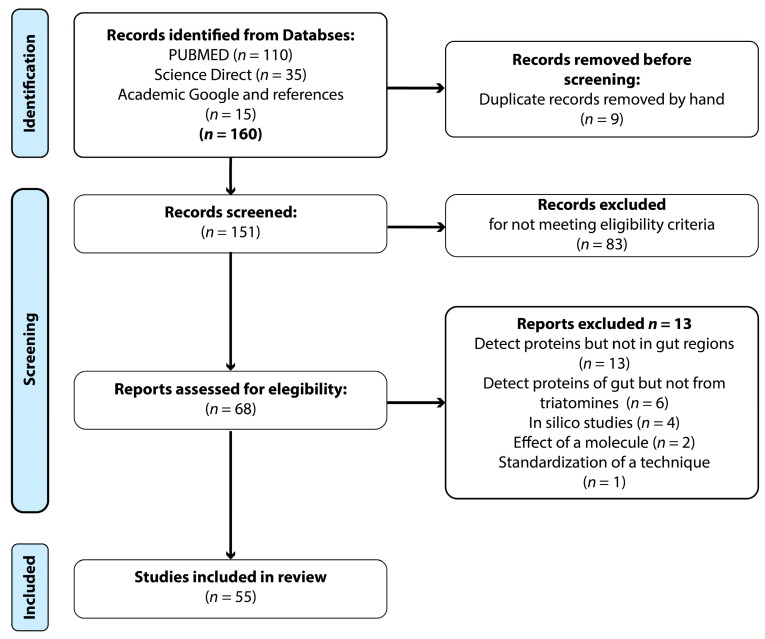
Article selection, based on the inclusion criteria.

From the one-hundred-and-forty-eight original research articles retrieved, three duplicates were excluded. After reading the titles and abstracts, 92 studies were removed because they did not meet the inclusion criteria. From the remaining 68 articles, 13 were removed, including some that had detected proteins but not in the triatomine gut (*n* = 6). Others were eliminated because they were studies of hypothetical proteins, in silico, or whose sequences were inserted for expression in other organisms (*n* = 4). Articles where the effects of the addition of a molecule were determined (*n* = 2) or where only one technique was standardized (*n* = 1) were also excluded. Finally, 55 articles that met the criteria were included in this systematic review (Figure 1, Flow Chart).

### 3.2. Characteristics of Included Publications

**Figure 2:**
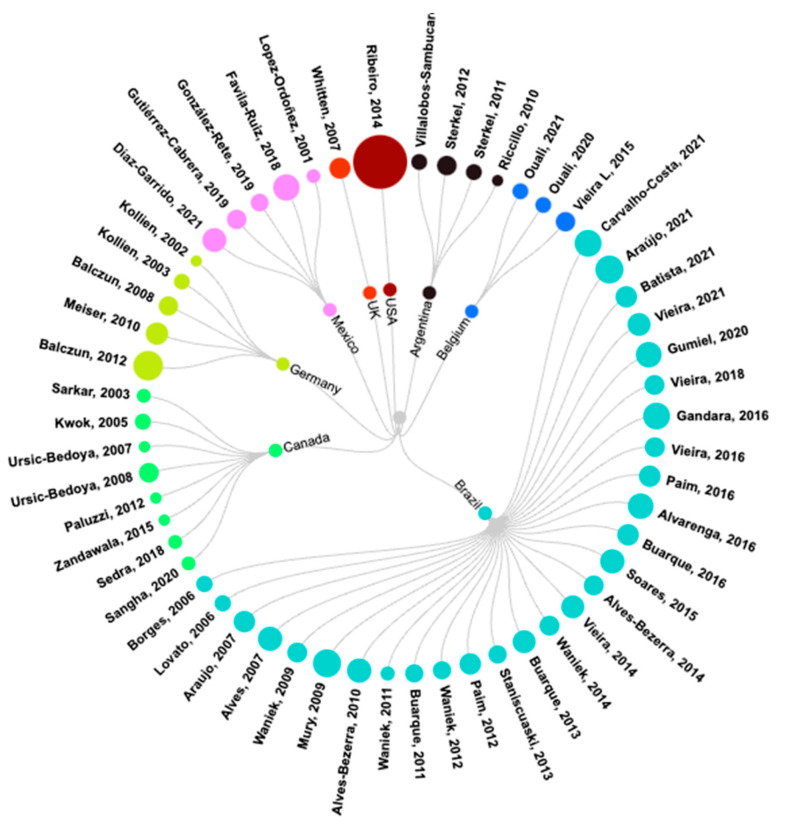
Publications reviewed, grouped by country of affiliation of the first author [7,12,15,16,17,18,19,20,21,22,23,24,25,26,27,28,29,30,31,32,33,34,35,36,37,38,39,40,41,42,43,44,45,46,47,48,49,50,51,52,53,54,55,56,57,58,59,60,61,62,63,64,65,66,67]. Dot diameter is proportional to the number of authors contributing to each publication. Circular dendrogram created with the visualization platform RawGraphs [68].

The studies reported in the 55 articles (see Table 1) were conducted in nine countries and involved a total of 245 authors. Brazil was the country of affiliation of the first author most frequently found, being associated with 55% of the publications, as shown in Figure 2. Most of the articles included six authors (mode = 6, variance = 37.2), and the studies were published in 30 different journals.

### 3.3. Reports of Transcripts, Proteins, and Glycoproteins

Transcript detection was reported in 42 of the 55 articles (76.36%) (see the Supplementary Table S1 of Transcripts Compendium), but only in 34 were they reported exclusively (61.81%). Eight articles reported transcripts and proteins in the same paper (14.54%). Proteins were reported in 21 papers (38.18%) (see the Supplementary Table S2 of Proteins Compendium), but only in 13 were they reported exclusively (23.63%). Among protein reports, only two studied glycoproteins (3.63%).

### 3.4. Triatomine Species Used in the Studies

**Figure 3:**
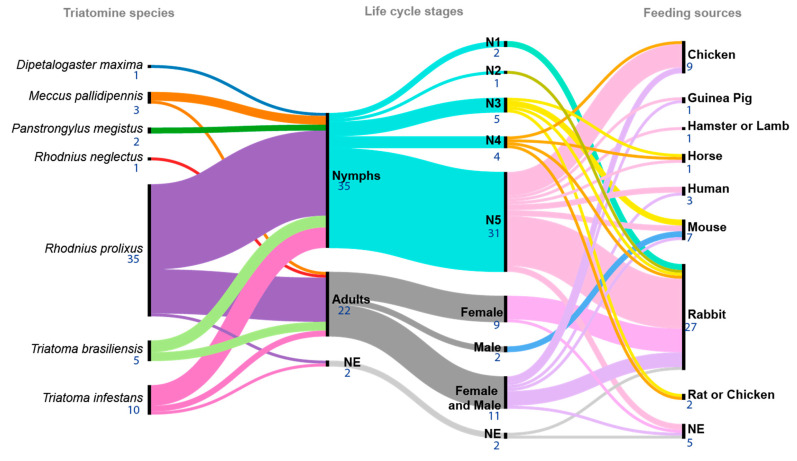
Triatomine species reported according to the life-cycle stages and food sources used in the 55 publications reviewed. In the left column, each triatomine species is indicated in a color: *D. maxima* (blue), *M. pallidipennis* (orange), *P. megistus* (green), *R. neglectus* (red), *R. prolixus* (purple), *T. brasiliensis* (light green), and *T. infestans* (bubblegum pink); the central column of life-cycle stages indicates whether each article reported nymphs (turquoise) or adults (dark grey); the right column indicates the feeding source of each specific nymph stage—N1 (mint green), N2 (apple green), N3 (yellow), N4 (light orange), N5 (blush pink), female (pink), male (sky blue), female and male (lilac), and NE (gray) when the life-cycle stage was not specified [7,12,15,16,17,18,19,20,21,22,23,24,25,26,27,28,29,30,31,32,33,34,35,36,37,38,39,40,41,42,43,44,45,46,47,48,49,50,51,52,53,54,55,56,57,58,59,60,61,62,63,64,65,66,67]. Under the species names, developmental stages, and food sources, the numbers of articles in which each condition was reported are shown. Alluvial diagram created with the visualisation platform RawGraphs [68].

Seven triatomine species were used as experimental models in the 55 articles (see the Supplementary Table S3 of Inclusion Criteria and Figure 3). The most frequent species was *R. prolixus*) (Stål, 1859) (*n* = 35), followed by *Triatoma infestans* (Klug, 1834) (*n* = 10), *T. brasiliensis* (Neiva, 1911) (*n* = 5), *Meccus pallidipennis* (Stål, 1872) (*n* = 3), *Panstrongylus megistus* (Burmeister, 1835) (*n* = 2), *R. neglectus* (Lent, 1954) (*n* =1), and *Dipetalogaster maxima* (Uhler, 1894) (*n* = 1). Only Gumiel et al. (2020) reported working with four triatomine species (*R. prolixus*, *P. megistus*, *T. infestans*, and *D. maxima*) [33].

With respect to triatomine life cycle stages, nymphal stages (*n* = 35 papers) and adult females and males (*n* = 20) were used in all experiments, with fifth-instar nymphs being the most common stage (*n* = 31). Female and male adults (*n* = 11), female adults (*n* = 9), third-instar nymphs (*n* = 5), fourth-instar nymph (*n* = 4), first-instar nymphs (*n* = 2), male adults (*n* = 2), and second-instar nymphs (*n* = 1) were also used. Two articles did not specify the developmental stage used (see the Supplementary Table S3 of Inclusion Criteria and Figure 3).

### 3.5. Feeding Conditions and T. cruzi Infection

The main feeding sources for the triatomines were rabbit (*n* = 27), chicken (*n* = 9), and mouse (*n* = 8) blood (see the Supplementary Table S3 of Inclusion Criteria and Figure 3). The use of an artificial feeding device was reported in 23 out of 55 articles; it was performed on live animals in 20, and it was not specified in 12.

A key variable is feeding with *T. cruzi*-infected blood, which was reported in 15 of 55 articles reviewed (see the Supplementary Table S3 of Inclusion Criteria). Nine strains/isolates were reported, most notably those in the Discrete Typing Unit (DTU) TcI, which were used in about half of the reports (53.3%, 8/15), in addition to DTU TcII in four (26.6%) and TcVI.

### 3.6. Triatomine Gut Regions

The 55 selected articles studied the guts of triatomines, which have three main regions: the anterior midgut (AMG), posterior midgut (PMG), and rectum (RE). The use of all three sections to perform experiments was reported in 23 articles; five of them reported to have pooled all three sections for analysis.

### 3.7. Analysis of Reported Transcripts

**Figure 4:**
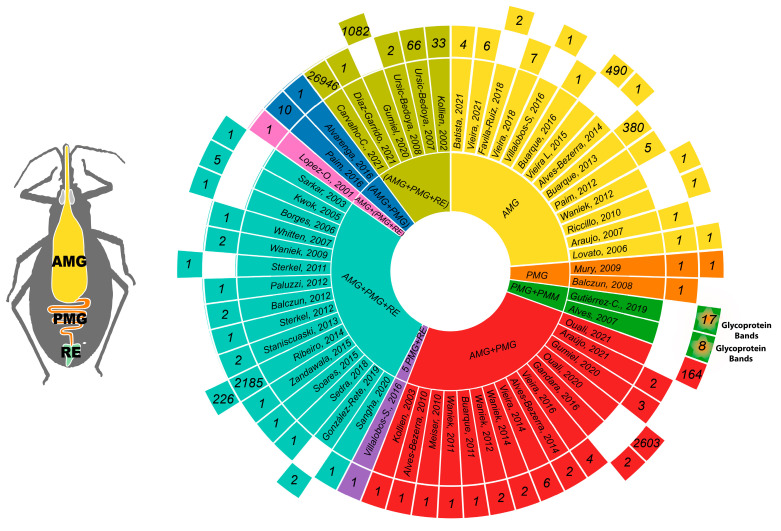
Transcript and protein detection in the different regions of the triatomine gut. Triatomine gut regions are shown in the central circle: AMG: anterior midgut (yellow), PMG: posterior midgut (orange), and RE: rectum; the use of two or more regions such as in PMG + PMM (green), AMG + PMG (red), PMG + RE (purple), and AMP + PMG + RE (turquoise); and combinations of a gut region and a pool of the gut regions indicated in parentheses, AMP + (PMG + RE) (pink), (AMP + PMG) (blue), (AMP + PMG + RE) (apple green). Publications reporting each gut section or combination are shown in the second circle. The numbers of transcripts and proteins reported per publication are shown in the third and fourth circle, respectively [7,12,15,16,17,18,19,20,21,22,23,24,25,26,27,28,29,30,31,32,33,34,35,36,37,38,39,40,41,42,43,44,45,46,47,48,49,50,51,52,53,54,55,56,57,58,59,60,61,62,63,64,65,66,67]. Sunburst diagram created with the visualisation platform RawGraphs [68].

**Figure 5:**
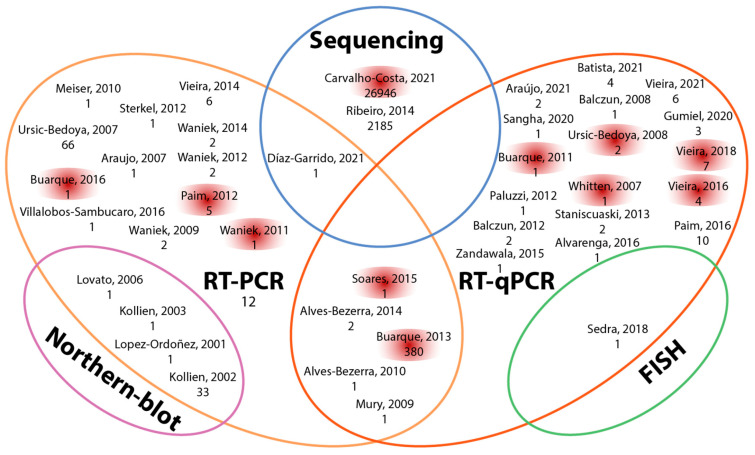
Techniques reported for transcript analysis in 42 articles. The last name of the first author, year of publication, and number of transcripts reported are shown for each article [7,12,15,16,17,19,21,22,23,24,25,26,27,28,29,30,31,32,33,34,35,37,38,39,40,41,42,44,45,47,49,50,52,53,54,56,58,59,60,61,62,63,64,66,67].

Forty-two articles collectively reported the detection of 29,692 transcripts in the triatomine gut (see the Supplementary Table S4 of Transcripts). Most articles analyzed fewer than 10 transcripts (*n* = 36); two articles reported 10–66 transcripts, and the remaining three papers reported the highest numbers of transcripts: Buarque (2013) reported 380 transcripts (1.3%) in the AMG of *T. infestans*; Ribeiro (2014) reported 2185 (7.4%) in the different sections of the gut of *R. prolixus*; and Carvallo-Costa (2021) reported 26,946 transcripts (90.8%) containing coding sequences in a pool of the three sections of the gut of *R. neglectus* [16,28,39] (Figure 4). For transcript detection, the articles reviewed used Northern blot, FITC, RT-PCR, RT-qPCR, and sequencing. The most common methods were RT-PCR and RT-qPCR, reported in 23 and 22 articles, respectively; however, the actual sequencing techniques used by Ribeiro and Carvallo-Costa allowed the detection of a larger number of transcripts [16,28] (Figure 5).

### 3.8. Analysis of Reported Proteins

The 21 articles identifying proteins in the guts of triatomines reported 4584 proteins in total. Of these, 155 were mentioned in the text of 18 articles, and these are the ones discussed in this review. The remaining three articles made no mention of proteins in the main text; for example, Ribeiro et al. (2014) listed them in a supplementary table [28]. Gutiérrez-Cabrera et al. (2019) and Alves et al. (2007) reported the molecular weights of lectin-recognized glycoproteins without identifying them [12,15].

**Figure 6:**
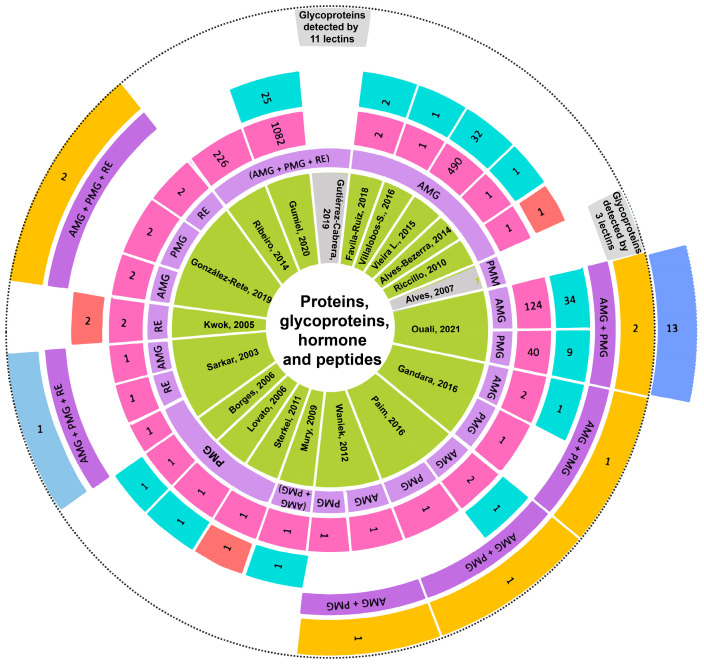
Proteins, glycoproteins, hormones, and peptides from the whole guts of triatomines that were mentioned in the text of 21 articles. The central circle, in green, shows the first authors and the years of publication of articles reporting proteins. The gray sector corresponds to papers that reported proteins and glycoproteins. The second circle, in lilac, shows, from the center outwards, the gut sections that each paper processed: AMG, PMG, RE, PMM, AMG + PMG pool, and AMG + PMG + RE pool. The third circle, in pink, shows the number of total proteins reported in each paper. The fourth circle, in turquoise, shows the number of proteins described in each text, and the sector in red shows the peptides. The fifth circle, in purple, shows the detection of the same proteins in combined sections: AMG + PMG and AMG + PMG + RE. The sixth circle, in yellow, shows the number of shared proteins; the sector in sky blue shows the hormones, and the gray sector shows the number of lectins used in the detection of glycoproteins. The dark-blue overhanging tab shows the 13 proteins reported by proteomic studies without prior reports in genomic databases [7,12,15,18,20,23,28,31,33,36,43,45,46,48,51,55,56,57,61,65].

A compendium of total proteins and those reported in the text of each article is listed herein. Reports of 1, 2, 164, 226, 490, 1082, and up to 2603 total proteins were found. The name of each protein was listed in the supplementary tables of each original article. URLs to these tables are shown in the Supplementary Table S2 of Protein Compendium in this review. Interestingly, the paper by Ouali et al. (2020) is the only one to report 13 proteins described for the first time, with no previous record in a genomic database [36] (Figure 6).

**Figure 7:**
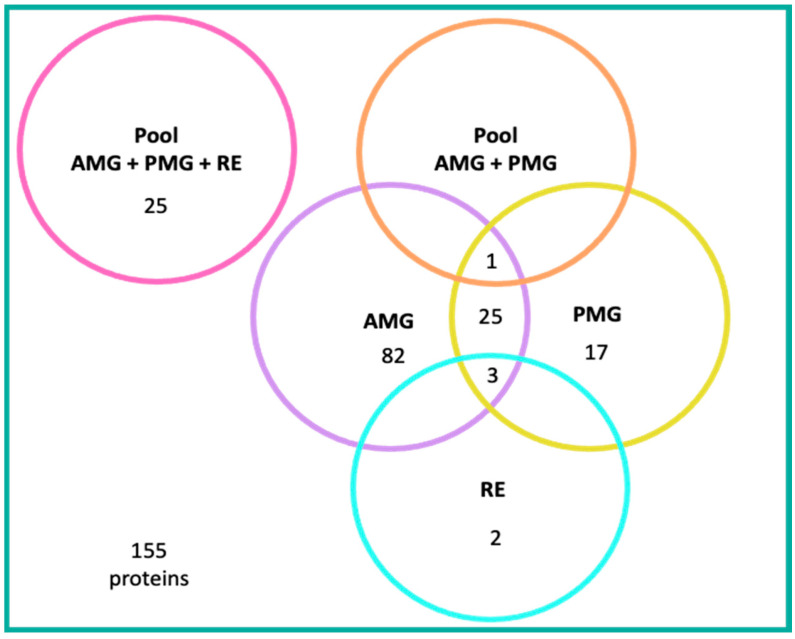
Number of proteins mentioned in text, by gut region, in 18 articles, from a universe of 155 proteins. Gut regions and pools are represented in circle shapes as follows: AMG + PMG + RE pool (pink), AMG + PMG pool (orange), AMG (lilac), PMG (yellow), and RE (turquoise).

Of the proteins mentioned in the text of the reviewed articles, 110 were reported in the AMG, 82 of which are unique to this gut section, including one peptide. Twenty-five are shared with the PMG, three with the PMG and RE, and one with an AMG/PMG pool [7,23,36,43,46,55,56,57,61] (Figure 6 and Figure 7).

Forty-five proteins expressed in the PMG were mentioned; 17 of them were exclusive to this gut section, including one peptide. The remaining 25 proteins were also expressed in the AMG, three in the PMG and RE, and one in an AMG/PMG pool [7,23,36,43,45,48,55,56,57,61,65] (Figure 6 and Figure 7).

Five proteins expressed in the RE were reported, two of which are unique to this gut section, whilst the remaining three are shared with the AMG and PMG [43,51,57] (Figure 6 and Figure 7).

The authors of two articles worked with whole-gut pooled samples (AMG, PMG, and RE). Gumiel et al. (2020) reported the expression of 25 proteins, and Gutierrez-Cabrera (2019) reported an electrophoretic profile with 17 bands in a wide molecular weight range (Figure 6).

**Figure 8:**
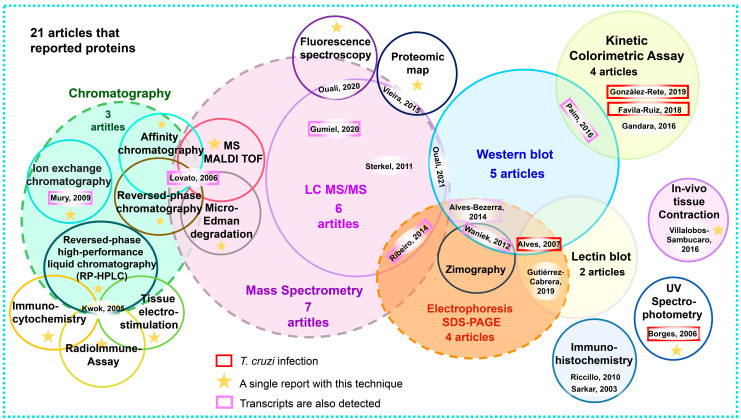
Techniques used in the 21 articles that reported proteins and glycoproteins. Each circle shows a technique. The circle diameters correlate to the numbers of articles used, and the numbers inside the circles correspond to each article listed in the Table S2 of Protein Compendium. Red boxes show the articles that worked with *T. cruzi*-infected triatomines. Lilac boxes show articles in which both proteins and transcripts were identified, and stars indicate techniques that were reported only once for protein detection [7,12,15,18,20,23,28,31,33,36,43,45,46,48,51,55,56,57,61,65].

Twelve experimental procedures were used for protein detection and analysis. Sub-variants of mass spectrometry, electrophoresis, and chromatography were used, for a total of 20 specific techniques (see Figure 8 and the Supplementary Table S2 of Protein Compendium).

The articles with the highest number of proteins reported were the most recent ones, which used systemic biology approaches to identify complex mixtures by using proteomic techniques and MS analysis [7,20,28,33,36]. The remaining papers characterized 1–2 proteins each [12,18,23,31,43,45,46,48,51,55,56,57,61,66] (Figure 8).

Some proteins were detected by using a single technique [15,18,23,43,46,48,55,57,65,66], whilst up to four complementary techniques were used in combination to identify, quantify, detect the activity of, and demonstrate the presence of certain proteins [51] (Figure 8).

MS was the most widely used technique for protein detection. It was used in seven of the twenty-one articles reviewed. Six of these papers used high-performance liquid chromatography coupled to mass spectrometry (LC-MS/MS) [7,20,28,33,36,65]. In three of these six articles, complex mixtures of protein extracts were analyzed by using a shotgun method [7,33,36]. Two sub-proteomes were analyzed. The first one was derived from bands obtained by the SDS-PAGE electrophoresis of soluble and membrane proteins expressed in the AMG [28]. The second one was the analysis of 476 protein spots extracted from the only published proteomic map of the AMG of *R. prolixus*, from which 490 proteins were identified [20]. Of the six papers that used LC-MS/MS, four works supplemented the analyses with other techniques to detect specific proteins [7,20,28,33]. The remaining two articles employed MS: Gumiel et al. (2020) reported 1082 proteins, while Sterkel et al. (2011), despite employing nano-LC-ESI, only reported one protein [33,65] (see Figure 8 and the Supplementary Table S2 of Protein Compendium).

Lovato et al. (2006) characterized infestin1R, which has inhibitory specificity for proteases, using four techniques: reversed-phase affinity chromatography, matrix-assisted laser desorption/ionization (MALDI-TOF), and Edman degradation [45] (see Figure 8 and the Supplementary Table S2 of Protein Compendium).

Proteins with enzymatic functions were identified in six papers by using spectroscopic measurements [36,43,46,55,61]. Four papers determined the presence and enzymatic activity of these proteins by using colorimetric kinetics [43,46,55,61]. In the remaining two articles, the presence of cathepsin D was evidenced by fluorescence [36] and ultraviolet [48] spectrophotometry (see the Supplementary Table S2 of Protein Compendium and Figure 8).

SDS-PAGE electrophoretic separations of complex mixtures [7,12,31,56,61] and protein recognition by Western blot (WB) with specific antibodies and/or lectins [12,15,28,31,56] were conclusive tools for protein detection in five articles (see Figure 8 and the Supplementary Table S2 of Protein Compendium).

The separation of protein extracts by affinity, ion exchange, and reverse-phase chromatographic techniques allowed the identification of 1–4 proteins in three articles [23,45,51] (see Figure 8 and the Supplementary Table S2 of Protein Compendium).

In an in vivo study on tissue contraction, Villalobos-Sambucaro et al. (2016) identified an allatostatin-C (AST-C) receptor that is expressed in the AMG and regulates contraction during feeding [66] (see Figure 8 and the Supplementary Table S2 of Protein Compendium).

Radioimmunoassay, immunocytochemistry, and tissue electrostimulation analyses were complemented by reverse-phase chromatography to identify two neuropeptide isoforms of tachykinins in *R. prolixus* [51] (see Figure 8 and the Supplementary Table S2 of Protein Compendium).

## 4. Discussion

### 4.1. Transcripts Are More Frequently Reported Than Proteins

The available information on transcripts and proteins detected in the digestive tracts of Chagas disease vectors was analyzed in this review. It was found that of the 55 original research articles analyzed, 42 papers had focused on the detection of transcripts and 21 on proteins.

This could be explained by the development of genetic studies, which laid the foundation for proteomic studies. In 1970, two-dimensional electrophoresis (2DE) marked the beginning of protein databases, which boosted these studies [69]. Subsequently, mass spectrometry became a powerful tool for the study of proteins. The complete sequencing of specific genomes marked the dawn of a new era, that of the functional analysis of gene products or so-called “functional genomics.”

### 4.2. Triatomine Species

About 150 species of triatomines have been reported in the world [70]; however, only a few species are considered competent vectors of *T. cruzi*. The 55 articles reviewed here do not reflect the diversity of species considered vectors, as only seven species were studied, among which *R. prolixus*, one of the main vectors of Chagas disease, was the most common. Interestingly, this species has reportedly been eradicated in El Salvador, Guatemala, Mexico, Nicaragua, Costa Rica, and Honduras [71]. It is noteworthy that no reports were found on *Triatoma dimidiata*, currently a major vector with active transmission in Central America and southeastern Mexico [70].

### 4.3. Discussion on Transcripts

#### 4.3.1. Transcripts by Gut Sections

Several processes that enable nutrient digestion and absorption, as well as parasite reproduction and differentiation, take place in triatomine gut sections; therefore, transcript and protein expression should show this discrepancy along the digestive tract, which varies depending on food intake and/or infection with different *T. cruzi* strains.

Of the 42 articles reporting the presence of transcripts in the digestive tract of triatomines, five used whole gut samples, in which a total of 27048 transcripts was detected (see the Supplementary Table S1 of Transcript Compendium) [16,19,32,35,60].

The detection of 2426 transcripts in the AMG was reported in 32 articles; 1982 of these transcripts were reported by Ribeiro [28] in supplementary tables. A differential expression of transcripts encoding for proteins with diverse functions was reported (see the Supplementary Table S4 of Transcripts), including mucins, cytoskeleton proteins, digestive enzymes, and peptidases. Among the peptidases, cathepsin B [28], cathepsin D—which was detected mainly in PMG [47]—and cathepsin L were remarkable; however, there are conflicting reports on the latter, as it was observed by Waniek [37], whilst Lopez-Ordoñez and Ribeiro failed to detect it in the AMG [28,63]. Regarding antimicrobial peptides, the increased expression of defensins A, B, and C transcripts was found [27,34,52,58], as well as of defensin 1 and 4 [26,62]. Other transcripts whose functions are associated with defense against other organisms are lysozymes; lysozymes A and B were reported to be more highly expressed in the AMG by Kollien and Vieira [34,54]. In contrast, Ribeiro was unable to detect lysozyme C in the AMG [28].

A total of 2056 transcripts detected in the PMG were reported in 25 articles. Ribeiro submitted 1982 of these transcripts, associated with diverse functions. Regarding antimicrobial peptides, the increased expression of prolixicin transcripts was detected in the PMG [34]. Defensins A, B, and C [27,34,52,58], as well as defensin 1 and 4 [26,62], have been found, although with lower expression in the PMG than in the AMG, unlike defensin 3, which was only detected in the PMG [26]. The peptidases cathepsin B and cathepsin L are among the transcripts showing higher expression in the PMG than in the AMG [28]; in fact, the latter was not detected in the AMG. On the other hand, the expression of cathepsins D1 and D2 was higher in the PMG [47]. Of note was the higher expression in the PMG of annexins 1, 2, and 3, whose regulation is associated with NOS induction by bacterial LPS [33]. The expression of the acyl-CoA-binding protein (RpACBP-1), which is a substrate for several metabolic processes and participates in cell signaling, is also higher in the PMG [21]; similarly, the neuropeptide receptor Rhopr-FGLa/AST-R was reported to be expressed more in the PMG than in the AMG and RE [25].

A total of 1955 RE-expressed transcripts were reported in 12 articles; 1941 of these transcripts were listed by Ribeiro et al. [28]. The transcripts that are chiefly expressed in the RE encode for products with diverse functions, from cytoskeleton structural proteins like actin to metabolic processes, transcription machinery, and protein synthesis. It is noteworthy that the expression of peptidases and digestive enzymes, as well as those associated with the immune response, was lower in this gut section than in the AMG and PMG.

#### 4.3.2. Feeding Stimulates Transcript Expression

Differences in transcript expression in the triatomine gut after feeding were analyzed in 12 articles (see the Supplementary Table S4 of Transcripts). Carvalho-Costa reported a significant increase in 24 transcripts in a whole-gut pool. The functions associated with these transcripts are diverse and include peptidases, products linked to oxidative metabolism, and translation factors [16]. Other studies searching for transcripts with specific functions found an increase in the expression of products associated with immunity, such as the immune deficiency gene (IMD) and RpRelish [61]; with defense against pathogens, such as lysozyme [34,54,61]; and with antimicrobial peptides, such as defensin B, prolixicin [34,61], defensin 3, and defensin 4. The latter was not detected under fasting conditions [26]. Transcripts associated with stress response, such as HSP70, endoplasmic reticulum (ER) stress genes, and other chaperones were also found [61]. Similarly, transcripts linked to proteolytic functions were detected, including cathepsin D (TiCatD) [47], S10 peptidase, and AA peptidase [16]; NO synthase (NOS) was also found [42], as well as products involved in the activation of antioxidant systems to protect midgut cells from oxidative damage (ROS) caused by blood digestion, such as RpUcp4 [31]. Other transcripts whose expression was increased after feeding were alpha-glucosidase [23] and acyl-CoA-binding protein (RpACBP-1) [21]. Some peptides did not show significant changes in their post-feeding expression, including peptidases such as cathepsin D2 (TiCatD2) [47], cathepsins L-1 and -2 (tbcatL-1 and bcatL-2), serine carboxypeptidases (tbscp-1 and tbscp-2) [37], defensin A [34], the uncoupling protein 5 (RpUcp5), chitin synthase [31,64], HSP90, ER stress genes, and the 14-3-3 epsilon protein gene (4-3-3ε) [61]. Finally, a decreased expression of aquaporin MIP variant A (RhoprMIP-A) [41], the antimicrobial peptide defensin C [34], HSP 69, NADPH-P450 reductase, and nine other transcripts was reported by Carvalho-Costa [16]. The expression of several transcripts is altered after feeding, and different works have sought to determine the presence of transcripts coding for proteins with specific functions expected to occur during digestion; however, no significant increase in the expression of these transcripts was observed in broader transcriptomic studies.

#### 4.3.3. *T. cruzi* Modulates Transcript Expression Differentially Depending on the Strain

Triatomine infection by *T. cruzi* has been shown to modify transcript expression. In the reviewed works (12), 40 different transcripts were detected, and the increased expression of 30 was reported; of these, 18 were found in the AMG of *R. prolixus* [39], four in a digestive tract pool of *R. neglectus* [16], and the rest in other works aiming to detect specific peptides. Among the most studied transcripts under infection conditions are those associated with defense against pathogens and antimicrobial peptides. An increased expression of transcripts of the TiAP-antimicrobial protein was found in the AMG of *T. infestans* [17], as well as of lysozyme A and B (RpLys-A and RpLys-B) in a gut pool of *R. prolixus* [32] and defensin 1 in T. *brasiliensis* infected with a strain isolated from the same vector (TBRA/BR/1999/JCA3) [62]. When determining the presence of antimicrobial peptides in *R. prolixus* infected by two different strains (TcI-Dm28c and TcII-Y), the expression of defensin A was found to decrease with both strains [58]. There was an increase in the expression of B defensin, C defensin, and prolixicin in insects infected with the TcI-Dm28c strain [52,58]; however, those infected with the Y strain did not show a significant change in C defensin and prolixicin; instead, the expression of B defensin decreased and Tigutcystatin showed increased expression [58,59]. This suggests that changes in the expression of transcripts in the guts of triatomines not only depend on the presence or absence of the parasite, but that there is a specific interaction that may allow the parasite to modulate protein expression. However, comparative studies are very limited and do not enable us to generalize this conclusion.

Some transcripts do not show significant changes in their expression, especially those coding for constitutive proteins such as glyceraldehyde-3-phosphate dehydrogenase, the 60S ribosomal protein, α-tubulin, and β-actin [50]. However, actin is one of the proteins whose expression Buarque found to be higher in infected individuals [39]. This finding should be taken with caution since, among other factors, both studies used different triatomine species and *T. cruzi* strains.

### 4.4. Discussion on Proteins

#### 4.4.1. Proteins Detected in the AMG

In the main text of 14 articles, 136 proteins were reported to be detected in the AMG; of these, 82 were exclusive to this gut region; 48 of these proteins were found in fed triatomines, 2 were found in infected insects, and the rest were unspecified (see the Supplementary Table S5 of Proteins Mentioned in Text). Of the AMG-exclusive proteins, 33 increased their expression levels in fed specimens [7,31,55] while the expression of 44 proteins did not change [18,20,36,61,66] (see the Supplementary Table S5 of Proteins Mentioned in Text).

Most of the proteins reported in the AMG were antioxidant and detoxifying enzymes (18.38%), heat shock proteins (11.02%), proteases (10.29%), oxidoreductases (7.35%), immune response-linked proteins (6.61%), and aspartyl proteases (4.4%). The rest of the proteins were described in lower frequencies and amounts, but together they constitute 41.96% of the total.

Among the proteins involved in the immune response of triatomines, lysozyme, an antimicrobial enzyme that degrades the cell wall in bacteria and regulates microbiota reproduction, especially in the AMG, is particularly interesting. Vieira et al. [20] found it in the AMG, and Ouali et al. [7] detected it in the same region and proved that its expression levels are increased six hours after feeding. This suggests that feeding and an overabundance of bacteria and/or parasites in food stimulate the expression of this enzyme in the AMG.

Another example is HSP70, a protein associated with heat stress and the correct folding of other proteins. Paim et al. [61] found this protein in the PMG, whilst Ouali et al. [7] reported that its expression levels in the AMG increased in fed insects. The AMG is the first site of contact between the warm blood of a mammal and the intestinal epithelium of the vector. This drastic increase in temperature triggers an immediate response, which would explain the increased expression of heat shock proteins in that region to maintain a controlled environment [7,61].

Three proteins decreased their expression in the AMG in fed vectors: NADPH-cytochrome P450 reductase, glutaredoxin, and the peptidase A1 domain-containing protein. However, the cause of this decrease is unknown since they are associated with the protection of the gut epithelium from oxidative stress [7].

These examples show that food intake triggers the differential expression of specific proteins in the AMG, which is the region that stores food after ingestion and before digestion.

#### 4.4.2. Proteins Detected in the PMG

In the PMG, 71 proteins were reported in 12 articles; 17 of these proteins were unique to that gut region [7,7,23,33,43,45,48,56,57,61,65] (See the Supplementary Table S5 of Proteins Mentioned in Text). Most of proteins detected in the PMG have detoxifying and antioxidant functions (18.3%), including heat shock proteins (HSP) (16.9%), oxidoreductases (14.1%), proteases (14.1%), immune response (enzymes) (5.63%), hydrolases (5.63%), isomerases (4.22%), and transferases (4.22%). The rest of the proteins were described in lower frequencies and amounts, but altogether they make up 16.90% of the total.

Of the seventeen proteins unique to the PMG, seven increased their expression levels after feeding (D-3-phosphoglycerate dehydrogenase; putative sulfotransferase; putative multicopper oxidase; multicopper oxidase; salivary platelet aggregation inhibitor 1; putative cathepsin l-like cysteine protease, and peptidase A1 domain-containing protein) [7], while cathepsin D did so after infection with *T. cruzi* [48]. On the other hand, only two proteins decreased their expression after feeding: glutathione S-transferase sigma class (GSTs4) and dihydrolipoyl dehydrogenase [7].

No changes were reported in the expression levels of the following PMG proteins after feeding: putative cathepsin l-like cysteine protease; putative cathepsin l-like cysteine proteinase; an uncharacterized protein; cytochrome P450 (putative cytochrome); cytochrome P450; and myosuppressin [36,65]. Feeding status was not specified for infestin 1R [45].

Most of digestion occurs in the PMG and is carried out by proteases. For triatomines, it is very important to produce large amounts of amino acids, as these are their source of nutrients [7,72]. Such is the case of aspartyl protease, an enzyme specifically associated with blood digestion and whose expression was increased in the PMG six hours after feeding [7]. Other works indicated that the sequence of this protease has 75% similarity with another aspartyl protease, called TiCatD, which is temporally expressed in the PMG of *T. infestans* after feeding, making it another example of feeding-dependent regulation [47]. Other proteins linked to digestion in the PMG are cysteinyl proteases (putative cathepsin l-like cysteine protease and putative cathepsin l-like cysteine proteinase) [36,63].

The expression of dihydrolipoyl dehydrogenase decreased in the PMG while it was increased in the AMG. This protein with antioxidant capacity prevents and reduces oxidative stress caused by blood intake, which suggests that its presence in the AMG is associated with the large amounts of stored blood therein. Its expression decreased in the PMG because the blood in this region is being processed and its amount decreases in time. This provides evidence that the presence of dihydrolipoyl dehydrogenase is substrate-dependent, and it has a great capacity to differentially regulate oxidation six hours after feeding [7].

#### 4.4.3. Proteins Expressed in Both AMG and PMG

A total of twenty-six proteins were detected in the AMG and PMG; of these, only two (putative HSP90 protein and a member of the HSP70 family) increased their expression in both regions after feeding. This demonstrates again the relevance of heat stress control after blood intake and digestion [7]. A catalase described by Gandara et al. [55] showed higher activity in the PMG than in AMG in non-fed organisms. Upon food ingestion, this protein was activated, and its levels were increased by the presence of reactive oxygen species (ROS) and by heat stress from ingested blood. Interestingly, 23 proteins did not show changes in their expression levels in either the AMG or PMG.

#### 4.4.4. Proteins Detected in RE

Four papers found 30 proteins in the RE, and only two isoforms, locustatachykinin I and locustatachykinin II, were reported to be exclusive to this gut region [23,33,36,43,51,56,57,61] (see the Supplementary Table S5 of Proteins Mentioned in Text). These neuropeptides have a dose-dependent stimulatory effect on intestinal muscle contraction in the RE, which could influence the processing of digested blood and the excretion of products. On the other hand, Sarkar et al. (2003) found a peptide called allatostatin, which they classified as a neurohormone, in all gut regions of *R. prolixus*. Contrary to the effect of locustatachykinins, allatostatin inhibited muscle contraction in the gut. A higher expression of this protein has been linked by immunohistochemistry to older and fasting insects.

The proteins detected in the RE were functionally classified as oxidoreductases (33.33%), hydrolases (13.33%), isomerases (10%), transferases (10%), immune response enzymes) (6.67%), innate immune system (6.67%), lyases (6.67%), neuromodulators that activate muscle contraction in the RE (6.67%), regulatory neurohormones/hormones on the hindgut (3.33%), and heat shock proteins (3.33%).

#### 4.4.5. Proteins Detected in Pooled Samples

A total of 25 proteins was detected in a pooled sample of the three regions; however, the feeding status of the triatomines used was not specified [33] (see the Supplementary Table S5 of Proteins Mentioned in Text). Regarding their functional classification, hydrolases, proteins of the innate immune system, isomerases, and oxidoreductases were reported. A disadvantage of working with a pooled sample is that the specific gut region for the expression of each protein is unknown.

#### 4.4.6. Food Stimulates Protein Expression

Eight papers reported 51 proteins whose expression levels increased; of these proteins, 48 were overexpressed after feeding and infection, and three were overexpressed under fasting conditions [7,31,43,46,48,51,55,57]. The functional classification of these proteins was very varied: proteases and protease inhibitors were included, as well as proteins related to detoxification/antioxidation, the immune response, lipocalins, amino acid metabolism, and neurohormonal/hormonal regulators, among others. Interestingly, catalase and citrate synthase were expressed mainly when triatomines were fasting [55]. Ouali et al. [7] reported an increase in the expression levels of 40 proteins in at least one gut region in fed organisms, among which proteases and proteins related to detoxification and antioxidation, as well as lipocalins, were especially abundant. Another aspartic protease is the A1 domain-containing protein peptidase, whose expression was clearly increased with respect to unfed insects; this protein is involved in the digestion of blood components such as hemoglobin and albumin, and its expression seems to be activated in response to blood ingestion. Another example is the heme-binding protein, an antioxidant protein that binds to the heme group derived from the digestion of hemoglobin. This suppresses the generation of reactive oxygen species and protects the intestinal epithelium of insects from oxidative stress during feeding [23].

Ouali et al. [7] also reported a decrease in the expression of five proteins in two gut sections: NADPH-cytochrome P450 reductase, glutaredoxin, and the peptidase A1 domain-containing protein in the AMG and glutathione S-transferase and dihydrolipoyl dehydrogenase in the PMG. The function of these proteins is related to protease activity and detoxification/antioxidation. For example, NADPH-cytochrome P450 reductase generates ROS via NADPH oxidation, and glutaredoxin has an important role in maintaining intracellular thiol-redox homeostasis by scavenging ROS. Therefore, the depletion of these proteins prevents heme-induced oxidative stress after feeding [55].

Finally, 99 proteins reported in nine articles failed to show significant changes in their expression levels after feeding. Seventy-four proteins were identified in a specific region of triatomine gut, and twenty-five were detected in a whole-gut pool [18,20,23,33,36,45,56,61,65,66]. The functional classification of these proteins included antioxidant enzymes, aspartyl proteases, detoxification, heat shock proteins, and proteases.

The proteins detected in gut pooled samples included oxidoreductases such as catalase, superoxide dismutase, and glutathione peroxidase. These are oxidative enzymes, involved in the protection of the gut epithelium from hydrogen peroxide and oxidative stress generated by blood digestion [33]. Cathepsin D (aspartic protease) and L (cysteine protease), two hydrolases were found in the digestive epithelium, which are involved in the digestion process of triatomines. A differential increase in the expression of these proteins in the AMG and PMG was reported in articles working with specific gut sections under different conditions of feeding and *T. cruzi* infection. This suggests a proteolytic role in digestion, and possibly in modulating the parasite–triatomine interaction [7,20,33,56].

#### 4.4.7. Proteins with Unchanged Expression Levels

Among the proteins for which no changes in their expression level were found in at least one gut segment were the tyrosine aminotransferase isoform X1 and aspartate aminotransferase. Both transaminases are vital for an insect, as they are involved in the degradation of amino acids, which is essential for its survival [20].

#### 4.4.8. *T. cruzi* Modulates Protein Expression Differentially, Depending on the Strain and Gut Section

In the articles reviewed, four authors studied the effect of infection by *T. cruzi* [12,43,46,48]. González-Rete et al. infected *M. pallidipennis* nymphs with the strains ITRI/MX/12/MOR (Morelos strain) and ITRI/MX/14/CHIL (Chilpancingo strain) [43]. Favila-Ruiz et al. worked with the strain ITRI/MX/12/MOR [46]. Both authors reported a significant increase in the activity of prophenoloxidase and phenoloxidase in the AMG in infected insects. Both enzymes are key in the immune response against pathogens and their clearance [73,74]. Since the AMG is the anatomical region where parasite-carrying blood is stored, it is there that the first recognition between the parasite, the microbiota and the gut epithelium of the triatomine occurs. The ingestion of food is a stimulus that triggers the host’s defense mechanisms; these produce a hostile environment that leads to the almost-immediate death of 80% of the parasites [75,76]. The opposite happens in the PMG and RE, where prophenoloxidase and phenoloxidase activity decreases. The reduced enzymatic activity in these sections may be associated with a lower number of parasites and their anchorage in the PMM [43].

Cathepsin B (85% proteolytic activity) and cathepsin D (15% proteolytic activity) have been shown to be the major digestive proteases in the PMG of *R. prolixus* [72]. In this regard, Borges et al. [48], using epimastigotes of the Dm28c (TcI) strain, reported that infection is associated with an increase in cathepsin D activity only in the PMG of *R. prolixus*. No cathepsin D activity was detected in either the AMG or the RE. It should be noted that *T. cruzi* did not show cathepsin D-type enzymatic activity, and so, the activity detected in the PMG in infected triatomines occurs due only to enzymes produced by gut cells. Finally, Alves et al. [12] also proved, using the Dm28c strain of *T. cruzi*, that there are sites of recognition, interaction, and binding at the carbohydrate level between the parasites and the PMM.

#### 4.4.9. Other Proteins and Glycoproteins

Two authors reported the detection of proteins and glycoproteins by using SDS-PAGE, WB, and lectin blots [12,15]. These techniques allowed the identification of proteins by molecular weight and by glycosylation type.

Gutierrez-Cabrera et al. detected 12 total protein bands weighing 15–150 kDa in a whole-gut pool from fasted insects and a total of 17 glycoprotein bands of several molecular weights detected by eleven lectins. The glycosylation pattern varies according to the feeding status of the triatomine [15]. In fed insects, 15 bands weighing 20–190 kDa were resolved. As 40, 57, 90, 130, and 150 kDa bands are shared between fed and fasted organisms, these proteins could be constitutive of the digestive system. However, the 20, 25, 30, 30, 80, and 190 kDa bands are unique to fed triatomines, suggesting that these proteins are expressed after food intake, for only the 15 kDa band corresponded to non-fed triatomines (see the Supplementary Table S6 of Glycoproteins).

In addition to the findings in whole-gut samples, Gutiérrez-Cabrera et al. [15] found 11 bands weighing 17–150 kDa in the PMM. The banding pattern matched that of fed organisms at molecular weights of 20, 40, 90, 130, and 150 kDa. The 40, 90, 130, and 150 kDa bands could be due, as mentioned above, to constitutive components of the digestive epithelium.

Alves et al. [12] reported total PMM proteins weighing 13–97 kDa. Eight 13–47.7 kDa glycoprotein bands in PMM were resolved by using WB, and these were identified by radiolabeling proteins from the Dm28c strain of *T. cruzi* and their binding to PMM proteins from fed insects (see the Supplementary Table S6 of Glycoproteins).

Gutiérrez-Cabrera et al. [77] screened protein glycosylation with the lectins GNA, LCA, AAL, AOL, RCA, SNA, MAA, Con A, PNA, and WGA; Alves et al. (2007) used the last three lectins as well. In addition, Gutiérrez-Cabrera et al. (2014) used an antibody specific to glycan-epitopes (α1,3-fucose residues) that is unique to insects.

It is noteworthy that a higher number of glycosylated proteins were found in fed insects than in fasted ones. This suggests the relevance of feeding in the expression of these glycoproteins [77].

Such was the case of the binding of D-galactose and D-mannose by the lectin Con A, which recognized 13 glycoprotein bands weighing 36–170 kDa in the guts of fasting insects. The 90, 65, 60, 60, 55, and 40 kDa bands were highly glycosylated, and the 36 kDa band was unique to this condition. On the other hand, Con A detected 19 17–170 kDa glycoproteins in the guts of fed insects. Bands of 80, 54, 35, 34, 34, 27, 24, 20, and 18 kDa were unique to this condition [77]. This suggests that food intake stimulates the expression of these glycoproteins, which are uniquely expressed in each feeding condition. In the case of PMM, Con A recognized 11 bands weighing 27–90 kDa, where the former band is uniquely detected in the intestinal epithelium of fed organisms (see the Supplementary Table S6 of Glycoproteins).

The binding of N-acetyl glucosamine (GlcNAc) and N-acetylneuraminic acid (sialic acid, Neu5Ac, or NANA) by WGA in non-fed organisms generated bands of 38–150 kDa, where glycoproteins of 40, 50, and 54 kDa were the most highly glycosylated. Exclusive of this condition were the 38, 125, and 150 kDa bands. In contrast, fewer glycoproteins were recognized in fed animals than under fasting, with 20 and 57 kDa glycoproteins being the most abundant, whilst 20 and 35 kDa glycoproteins were exclusive to the guts of fed organisms [77]. Once again, it is clear that feeding modulates the expression of several glycoproteins and that this particular lectin led to a higher recognition in non-fed organisms. As for the PMM, 70, 90, and 130 kDa glycoproteins were recognized by WGA, but none were detected in unfed or fed triatomines (see the Supplementary Table S6 of Glycoproteins).

The monosaccharide Gal-β (1-3)-GalNAc was recognized with the lectin PNA in a 65 kDa band in both feeding conditions and in the PMM. This recognition evidenced a constitutive glycoprotein of the intestine, since it was present in any feeding condition and even in the PMM, which is synthesized in the presence of food [77].

Alves et al. (2007) reported the presence of glycoproteins in the PMM by binding with lectins. Four of them were recognized by Con A, PNA, and WGA. PNA- and WGA-bound proteins showed a banding pattern of 40.5, 44, and 45.5 kDa, whereas Con A-bound glycoproteins weighed 31, 40.5, and 45.5 kDa, the former being exclusive to the recognition of α-D-mannose, α-D-glucose, and high-mannose-type N-linked glycans [12] (see the Supplementary Table S6 of Glycoproteins).

Of particular note is the inhibitory selectivity of INF1R for subtilisin A, neutrophil elastase, and chymotrypsin. This was characterized in the midgut of a hematophagous insect. Therefore, INF1R could be important for food acquisition and storage and could be an adaptation to control dangerous enzymes from microorganisms in food.

#### 4.4.10. Recognition between Glycoproteins in Triatomine Gut and *T. cruzi*

Bonay et al. [9] reported that *T. cruzi* expressed specific carbohydrate-binding proteins (CBPs) that show a clear differential specificity toward glycans with high mannose content. These CBPs have a high affinity for mannose oligomers with a Man-α1-2-Man-α1-6-Man-α1-6 structure. In contrast, trypomastigote CBPs showed a 400-fold lower affinity for these larger oligomers and are slightly more specific for Man5Glc than for Man9GlcNAc. This would explain why epimastigotes bind to the PMM in the PMG to subsequently move to the RE and complete their differentiation to metacyclic trypomastigotes.

Alves [12] demonstrated that there is an interaction between epimastigote CBPs and PMM glycoproteins in *R. prolixus*. He detected 8 recognition bands weighing 13–47.7 kDa. Those of 31, 40.5, 44, and 45.5 kDa were recognized by three lectins. This may suggest that they are structural glycoproteins of the PMM. Furthermore, that author proposed that the presence of hydrophobic proteins in epimastigotes was important for interacting with PMM proteins. Finally, studies by Gutierrez-Cabrera et al. [15] also demonstrated a differential expression of glycoproteins in the intestine and PMM through selective lectin affinity to glycoproteins.

The evidence above supports the suggestion that there are specific recognition sites between sections of the insect gut and the epimastigote and trypomastigote stages of *T. cruzi* in order to continue its life cycle.

Thus, it can be concluded that the level of expression, affinity, abundance, and exclusivity in lectin-binding glycoproteins in different gut sections and the PMM is determined by the feeding and infection conditions of the insects in question. This could explain that the glycoproteins detected by Alves [12] and Gutiérrez-Cabrera et al. [77] were not the same, since Alves [12] fed vectors with human blood infected with *T. cruzi* DM28c; the insects in that study were euthanized at 10 days, and the PMM was examined. Gutierrez-Cabrera et al. [77] fed their vectors with uninfected rabbit blood, and the whole intestine was dissected and studied on day 15 post-feeding.

## 5. Conclusions

Only seven triatomine species have been studied in works related to the expression of intestinal transcripts and proteins in *T. cruzi* vectors, among which *R. prolixus* has been the most common one. Thus, a wide field of study is open to learn about the variability of transcript and protein expression in other vector species.

The functional and structural relationships established between *T. cruzi* and triatomines are products of their co-evolution. Triatomines are adapted to the different areas they colonize and have the molecular machinery to endure both long fasts as well as the ingestion of large volumes of food. They have signaling cascades to sense and synthesize transcripts, proteins, and glycoproteins differentially at each specific time point in their lives. In each gut section, they manage temperature stress (e.g., HSP70 and HSP90), the presence of excess hemoglobin (e.g., aspartyl protease and heme-binding protein), the clearance and/or combat of microorganisms such as *T. cruzi* (e.g., lysozymes [A and B], defensins [A, B, and C], and phenoloxidase), fasting conditions (e.g., catalases and cathepsin L-1 and -2 [tbcatL-1 and 2]), modulators and inhibitors of gut muscle movements (e.g., locustatachykinin [I and II] and allatostatin), the degradation of proteins to synthesize their own molecules (e.g., cathepsins [B, D, and L] and trypsin-like serine protease), and the growth and continuation of their life cycles (e.g., 60S ribosomal protein, α-tubulin, β-actin, and cytochrome P450) to ensure the permanence of their species.

Infection by different *T. cruzi* strains and/or isolates has been shown to modify the expression of transcripts, proteins, and glycoproteins in the triatomine gut. However, studies are limited and do not allow us to generalize this modulation, showing a very interesting information gap to explore.

The PMM is a vital structure for the establishment of the parasite in the PMG. It contains abundant glycoproteins that are recognized by carbohydrate-binding proteins of *T. cruzi* and allow it to establish, differentiate, and continue its life cycle.

Under this scenario, further studies should be conducted to determine still-unknown molecular mechanisms underlying the vector–parasite interaction, which would allow us to propose new approaches to interrupt the transmission of *T. cruzi* and reduce the incidence of Chagas disease.

## Figures and Tables

**Table 1:** Included articles. The 55 articles that met the criteria were included in this systematic review.

#	Year	Author	T/P	Ref.	#	Year	Author	T/P	Ref.	#	Year	Author	T/P	Ref.
1	2021	Carvalho-Costa, et al.	T	[16]	20	2016	Buarque, et al.	T	[17]	39	2010	Riccillo, et al.	P	[18]
2	2021	Díaz-Garrido, et al.	T	[19]	21	2015	Vieira L., et al.	P	[20]	40	2010	Alves-Bezerra, et al.	T	[21]
3	2021	Ouali, et al.	P	[7]	22	2015	Soares, et al.	T	[22]	41	2009	Mury, et al.	T&P	[23]
4	2021	Araújo, et al.	T	[24]	23	2015	Zandawala, et al.	T	[25]	42	2009	Waniek, et al.	T	[26]
5	2021	Batista, et al.	T	[27]	24	2014	Ribeiro, et al.	T&P	[28]	43	2008	Balczun, et al.	T	[29]
6	2021	Vieira C., et al.	T	[30]	25	2014	Alves-Bezerra, et al.	T&P	[31]	44	2008	Ursic-Bedoya, et al.	T	[32]
7	2020	Gumiel, et al.	T&P	[33]	26	2014	Vieira C., et al.	T	[34]	45	2007	Ursic-Bedoya, et al.	T	[35]
8	2020	Ouali, et al.	P	[36]	27	2014	Waniek, et al.	T	[37]	46	2007	Alves, et al.	P	[12]
9	2020	Sangha, et al.	T	[38]	28	2013	Buarque, et al.	T	[39]	47	2007	Araujo, et al.	T	[40]
10	2019	Gutiérrez-Cabrera, et al.	P	[15]	29	2013	Staniscuaski, et al.	T	[41]	48	2007	Whitten, et al.	T	[42]
11	2019	González-Rete, et al.	P	[43]	30	2013	Sterkel, et al.	T	[44]	49	2006	Lovato, et al.	T&P	[45]
12	2018	Favila-Ruiz, et al.	P	[46]	31	2012	Balczun, et al.	T	[47]	50	2006	Borges, et al.	P	[48]
13	2018	Sedra, et al.	T	[49]	32	2012	Paim, et al.	T	[50]	51	2005	Kwok, et al.	P	[51]
14	2018	Vieira C, et al.	T	[52]	33	2012	Paluzzi, et al.	T	[53]	52	2003	Kollien, et al.	T	[54]
15	2016	Gandara, et al.	P	[55]	34	2012	Waniek, P et al.	T&P	[56]	53	2003	Sarkar, et al.	P	[57]
16	2016	Vieira, C. et al.	T	[58]	35	2011	Buarque, et al.	T	[59]	54	2002	Kollien, et al.	T	[60]
17	2016	Paim, et al.	T&P	[61]	36	2011	Waniek, et al.	T	[62]	55	2001	Lopez-Ordoñez, et al.	T	[63]
18	2016	Alvarenga, et al.	T	[64]	37	2011	Sterkel, et al.	P	[65]					
19	2016	VillalobosSambucaro, et al.	T&P	[66]	38	2010	Meiser, et al.	X	[67]					

# corresponds to the ID number of the selected articles in ascending order starting with the most recent publication. Year of publication, first author, report of transcripts (T) and/or proteins (P), and bibliographic reference [7,12,15,16,17,18,19,20,21,22,23,24,25,26,27,28,29,30,31,32,33,34,35,36,37,38,39,40,41,42,43,44,45,46,47,48,49,50,51,52,53,54,55,56,57,58,59,60,61,62,63,64,65,66,67].

## Supplementary Materials

The following supporting information can be downloaded at https://www.mdpi.com/article/10.3390/pathogens12091124/s1—Supplementary Table S1: Transcript Compendium; Supplementary Table S2: Protein Compendium; Supplementary Table S3: Inclusion Criteria; Supplementary Table S4: Transcript; Supplementary Table S5: Proteins Mentioned in Text; Supplementary Table S6: Glycoproteins [7,12,15,16,17,18,19,20,21,22,23,24,25,26,27,28,29,30,31,32,33,34,35,36,37,38,39,40,41,42,43,44,45,46,47,48,49,50,51,52,53,54,55,56,57,58,59,60,61,62,63,64,65,66,71].
